# Extracellular Vesicles in Teasing Apart Complex Plant-Microbiota Links: Implications for Microbiome-Based Biotechnology

**DOI:** 10.1128/mSystems.00734-21

**Published:** 2021-08-24

**Authors:** Thabiso E. Motaung, Emma T. Steenkamp

**Affiliations:** a Division of Microbiology, Department of Biochemistry, Genetics, and Microbiology, University of Pretoria, Hatfield, Pretoria, South Africa; b Forestry and Agricultural Biotechnology Institute, University of Pretoria, Hatfield, Pretoria, South Africa

**Keywords:** extracellular vesicles, microbiome, microbiome biotechnology, plant-microbe interaction, plant immunity, biomarker, diagnostics, microbiota manipulation

## Abstract

Extracellular vesicles (EVs) are subcellular carriers of bioactive compounds with a complex array of functional effects on target cells. In mammals, circulating bodily fluid microbiota EVs (mbEVs) deliver cargo from source cells and adversely or favorably alter the physiology of the same source, neighboring, and distant recipient cells in an autocrine, paracrine, or endocrine fashion, respectively. Plant mbEVs may similarly mediate these interactive effects within the holobiont framework. However, the majority of plant EV research has focused on a small number of individual microbes, thus failing to reflect the importance of EVs in a community and consequently leaving a wide gap in scientific knowledge. Addressing this gap should entail a systems-level approach that combines vesicle characterization with microbiome analyses. This would certainly usher in a new age in microbial biotechnology entailing EVs as a microbiome manipulation strategy, a biomarker for stable microbiomes, and a diagnostic tool for plant infectious diseases.

## COMMENTARY

One of the most pressing agricultural challenges of the 21st century is improving crop yield and quality. Efforts to address this challenge vary but often entail the use of microbiota (i.e., microbial consortia of commensal, symbiotic, and pathogenic microorganisms). However, lifting the veil on microbial community dynamics to optimize plant production is not straightforward since diverse factors, including plant health and the environment, affect community composition. In turn, plant community relationships rely heavily on intricate systems for effective resource allocation and communication, one of which is embodied by extracellular vesicles (EVs).

EVs are lipid bilayer-enclosed nanoparticles of about 30 nm to several micrometers in diameter. They facilitate the intercellular transfer of molecules from source cells over short and long distances, which are then absorbed by recipient cells via membrane fusion and endocytosis. EV-based communication systems are well understood in mammals, where their release can be deregulated in diseased but not healthy cells (1), suggesting that EVs have tremendous therapeutic and diagnostic potential.

Because EVs tend to mirror their donor cell features and functions, albeit to various degrees (1), they are likely to have a lot in common with their donor microbiota. As a result, microbiotic EVs (mbEVs) could aid in a variety of advantageous outcomes in plants, including nutrient uptake, detoxification of harmful compounds, and the secretion of antimicrobials and volatiles that inhibit infection. The study of EVs from microbiomes is thus extremely relevant, especially given the intimate relationship between microbiota and plant host cells.

## BIOGENESIS, COMPOSITION, SUBTYPES, AND ROLES OF VESICLES

For decades, EVs have been perceived as nothing more than disposal bags of cellular debris, but recent findings suggest they are, in fact, multifunctional shuttles of a wide range of cargo destined to cross biological membranes (1). Cargo can be carried both on the cell surface as transmembrane factors (e.g., tetraspanins, integrins, and lipid rafts), and internally (e.g., proteins, polysaccharides, lipids, metabolites, and nucleic acids). EV production has been reported from all domains of life but is well understood in bacteria and eukaryotes. In bacteria, vesicles are generally released during bulging-out of the outer membrane, resulting in outer membrane vesicles. The same principle may apply during the release of eukaryotic EVs (microvesicles and apoptotic bodies) from plasma membranes (1). However, intraluminal vesicles emerge by reverse budding from late endosomal compartments, giving rise to EVs known as exosomes (1). Other types of EVs include a double membrane-bound exocyst-positive organelle identified in Arabidopsis thaliana and Nicotiana tabacum (2), pollensomes that were described originally from germinating Olea europaea pollen grains (3), Ms-vesicles produced by mycosomes of certain endophytic fungi during an apparent cell wall-less protoplast phase (4), aflatoxigenic vesicles (aflatoxisomes) involved in extracellular export of aflatoxin in Aspergillus parasiticus (5), exomeres, representing nonmembranous nanoparticles of tumor cell lines (6), and mitovesicles, representing vesicles associated with mitochondrial origin and dysfunction (7). This diversity in vesicle subtypes clearly demonstrates that EVs impart diverse functions and have a complicated subcellular origin.

EV functions stem from cargo internalized from source cells in a source-sink fashion, which is then delivered to recipient cells. As such, cargo loading is tightly regulated by the source cell (1), which can compositionally overlap the EVs it releases. However, factors such as uneven membrane invagination and genetics can result in selective cargo loading and compositional differences between EVs and their source cells. Moreover, EVs may be biased toward delivering certain types of cargos, such as in the case of small RNAs (sRNAs), whereby such bias is partly due to sRNA size, high abundance, frequent interaction with membranes, and cytoplasmic rather than nuclear localization (8). Various other RNAs are also carried by plant and fungal EVs during *trans*-kingdom bidirectional gene regulation (9), which is currently being exploited for practical purposes such as field protection of crops against and prevention of postharvest losses due to pests and pathogens (10, 11).

In addition to RNAs, other EV cargos have been identified, many of which promote microbial pathogenesis, virulence, and microbial competition (12). Additionally, some of the microbe-derived EV cargos are immunogenic and mimic microbe-associated molecular patterns (e.g., EF-Tu and polysaccharide A). As such, upon EVs making contact with host surface-localized pattern recognition receptors, these cargos can induce defensive immunity and EV-induced defense-related gene expression (13), which in turn keeps the plant immune system on high alert for future invaders. There are many similar instances where EV-associated cargos exert effects on host immune responses (12), hinting at the conserved immunomodulatory effects of EVs across microbial species. Therefore, there is a good chance that mbEVs also carry some immunogenic cargos in addition to cargo generally involved in plant-microbe interactions.

## VESICLES CONTRIBUTE TO PLANT-MICROBIOME FUNCTIONING

EV-mediated benefits to plant-microbiome functioning, some of which are briefly discussed in Table 1, can be uncovered primarily through EV isolation and characterization. EV composition may mirror the physiochemical impacts of microbiomes on plants, and its analysis can enhance our understanding of how microbial communities boost fitness and competitiveness over nutrients and favorable plant microniches remotely from EV donor microbes. A similar event occurs in mammals whereby circulating tumor-derived EVs remotely alter the behavior of neighboring cells to aid progression (14). Therefore, isolation and manipulation of biodistributed EVs from microbial communities can be a useful tool for studying diverse community functions in complex eukaryotic systems.

**TABLE 1:**
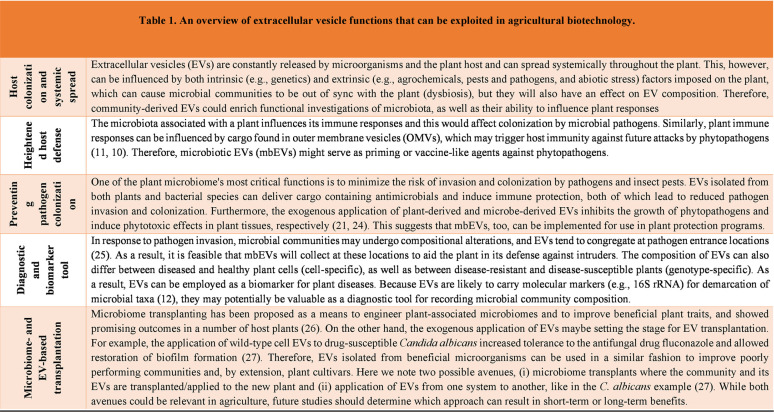
Overview of extracellular vesicle functions that can be exploited in agricultural biotechnology

EVs further facilitate beneficial communication between eukaryotic hosts and their microbiomes. For instance, colonic epithelial cell-derived vesicles facilitate the production of host-beneficial compounds by gut microbial communities (15). It is likely that mammalian mbEVs produced by gut flora microbes also distribute vital resources via body fluids to influence important host physiological functions (12). Indeed, microbe-derived EVs reportedly distribute vital resources within microbial communities, and this enhances certain virulence factors, including biofilm formation (16). This means that plants may similarly undertake EV-mediated communication with resident microbes. This was demonstrated with ginger-derived EVs that affect the composition and location of gut microbiota, as well as host physiological functions (17). EV-mediated communication also has been observed in microbes thriving at the extremes (18), suggesting communities containing these and apparently unculturable microbes, too, may launch EVs into plant tissues, thereby enabling remote modification of their microenvironments. This suggests that EV characterization is crucially relevant for studying microbiomes thriving at extreme environments, especially from unculturable microbial species presenting a significant hurdle to fully functionalizing microbiomes. The convergence of EV and microbiome analyses will therefore enrich our knowledge of mechanisms underlying plant-microbiota interaction.

## ENDOPHYTES AS A CASE STUDY IN PLANT-MICROBIOTA-VESICLE LINKS

Plants and endophytes have a mutually beneficial relationship, but precisely how EVs mediate this relationship is undocumented. We aim to address this gap in knowledge by investigating EV-mediated interactive strategies employed by plants and their endophytes, which is motivated in part by the following factors: (i) it is not novel to find EVs during plant-endophyte interaction, and in fact, several ultrastructural analyses have detected late endosomal EVs at the plant-endophyte barrier (19), which implicates EVs in the bidirectional *trans*-kingdom movement of cargo; (ii) mbEVs from endophytes colonizing the root apoplast can be isolated using protocols typically adapted for EV isolation from the leaf and root apoplast (20, 21); and (iii) these mbEVs will potentially coisolate with plant apoplastic EVs since their donor microbes also occupy the apoplast (e.g., 22). These mbEVs may contain molecular marker genes that enable microbial identification, such as 16S rRNA and internal transcribed spacer (ITS) regions (12). Indeed, microbial taxa have been successfully delineated up to the genus level using EV-associated 16S rRNA from biofluids (23), highlighting the utility of EVs in species identification. As a result, characterizing root-isolated EVs will allow us to identify endophytes participating in EV-mediated communication with plants. In this way, isolation of endophytic mbEVs represents an important step toward gaining functional insights into endophytes’ contribution to plant survivorship and the partners’ coevolutionary trajectories.

## EMERGING OPPORTUNITIES

Expanding upon the EV link between plants and their associated microbiomes for ultimately facilitating broad implementation of microbiome-based biotechnology in agriculture represents an intriguing opportunity. The extensive signal exchanges between microbial communities and plant host cells would certainly be reflected in community-released EVs. Therefore, integration of microbiome with EV studies would not only enhance microbial community profiling through molecular “omics” (e.g., genomics, proteomics, transcriptomics, and metabolomics), but would also fuel a cascade of agricultural applications (Table 1). This is particularly feasible since mbEVs characterized from microbiota can be manipulated for exogenous use by exploring a range of benefits, including using mbEVs as vaccines against plant infectious diseases, as priming agents for enhanced field performance, or as EV transplants for microbiome manipulation (Table 1). However, although mbEVs are worthy of examination, reliably forecasting the caveats and opportunities for their future in microbiome science is not yet possible. Also, before the application side of this proposition is in full swing, certain issues (e.g., reproducibility, precision targeting, scalability, and EV production formats) must be addressed. Nevertheless, the vast majority of studies daring to venture into plant-microbiota-EV links will contribute to unleashing the inherent microbial community potential. This would facilitate a dialogue between scientists across regional and international borders actively involved in the study of EVs and microbiomes to sustainably contribute to the quality of life using agricultural biotechnology.
